# Transportation for fuel: Thylakoid membrane bestrophin channels facilitate HCO_3_^−^ transport to the pyrenoid in diatoms

**DOI:** 10.1093/plphys/kiae133

**Published:** 2024-03-06

**Authors:** Jiawen Chen

**Affiliations:** Plant Physiology, American Society of Plant Biologists; Division of Crop Biotechnics, Department of Biosystems, KU Leuven, Leuven 3000, Belgium

Marine diatoms are unicellular photosynthetic organisms that are an essential part of the global ocean ecosystem. They are a phenotypically diverse group of phytoplankton, morphologically categorized into the round centric diatoms and elongated pennate diatoms (Alverson et al. 2006). Diatoms formed through secondary endosymbiosis, with their chloroplasts originating from red algae. As such, their chloroplasts have 4 membranes (Fig. A): the chloroplast endoplasmic reticulum, periplastidal membrane, outer chloroplast envelope, and inner chloroplast envelope. Thylakoid membranes sit within the chloroplast stroma, consisting of the girdle lamella and stroma thylakoid. At the center lies the pyrenoid, a membraneless organelle providing a CO_2_ concentrating mechanism (CCM), crossed by the pyrenoid-penetrating thylakoid (PPT).

**Figure.**
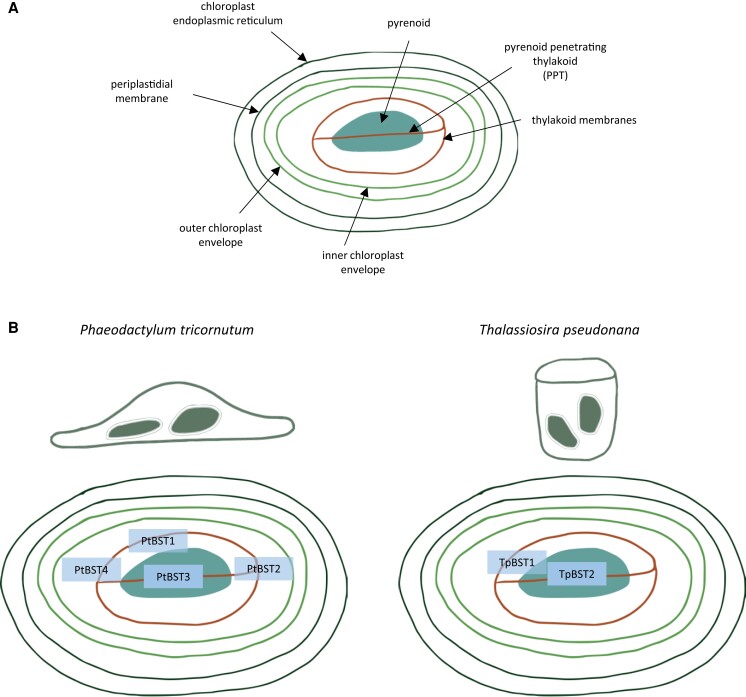
Diatom chloroplasts have BSTs with specific localizations. **A)** Schematic of the general structure of a diatom chloroplast with 4 membranes, with thylakoid membranes around the pyrenoid within. The thylakoid membrane is depicted as a single line for clarity but is made up of multiple layers of membranes. **B)** Summary of the localization of different BST channels studied in Nigishi et al. (2024) in the 2 species of diatoms. PtBST3 and TpBST2 were localized to the PPT in the pyrenoid, while PtBST1, 2, 4 were localized to the thylakoid membrane surrounding the pyrenoid. TpBST1 was localized both in the pyrenoid and throughout the thylakoid membranes. Transparent rectangles for the BSTs indicate localization found broadly throughout the stroma thylakoid membrane.

This CCM helps diatoms perform efficient photosynthesis in a seawater environment where CO_2_ levels are low. Most of the carbon here is in the form of HCO_3_^−^ (bicarbonate) and is imported by plasma membrane transporters (Nakajima et al. 2013), after which carbonic anhydrases located in the lumen of the PPT convert HCO_3_^−^ into CO_2_ (Kikutani et al. 2016; Nawaly et al. 2023). The converted CO_2_ is further concentrated around Rubisco in the pyrenoid to favor carbon fixation. However, it is still not clear exactly how diatoms facilitate the transport of HCO_3_^−^ across thylakoid membranes to the pyrenoid.

Bestrophins (BSTs) are membrane anion transporters that transport a range of ions, including Cl^−^ and HCO_3_^−^ (Qu and Hartzell 2008). In the green alga *Chlamydomonas reinhardtii*, 4 BSTs have been identified to transport ions across thylakoid membranes near the pyrenoid; BST1, BST2, and BST3 are the main HCO_3_^−^ transporters and are essential for maintaining growth at low CO_2_ concentrations (Mukherjee et al. 2019), while BST4 may be important in balancing the dynamics of the CCM and photosynthetic ion management during light to dark transitions. BST4 may function in transporting HCO_3_^−^ back from the pyrenoid into the thylakoid lumen (Adler et al. 2023). BSTs are therefore promising candidates for the HCO3^−^ transport across thylakoid membranes in diatoms but have not yet been studied. Convergent evolution likely resulted in the formation of CCMs and pyrenoids in both green algae and diatoms, but within this there is still a huge diversity in the forms that pyrenoids take among different organisms. Understanding how the diatom CCM compares to other known systems will greatly advance our understanding of carbon fixation in aquatic ecosystems.

In this issue of *Plant Physiology*, Nigishi et al. (2024) characterized 4 BSTs in the pennate diatom *Phaeodactylum tricornutum* and 2 BSTs in the centric diatom *Thalassiosira pseudonana*. These BSTs are localized to the chloroplast in specific patterns, some more associated with the PPT than others. The BSTs of both diatoms are upregulated at lower atmospheric CO_2_ concentrations. Under these conditions, a knockout mutant of PtBST1 had a lower growth rate, lower affinity for dissolved inorganic carbon (DIC) during photosynthesis, and higher nonphotochemical quenching, suggesting PtBST1 has roles in both the CCM and photosynthetic energy distribution.

While the authors found that BSTs are important for the CCM in both species of diatoms, they identified some differences between the BSTs in the 2 species. Using the Chlamydomonas BST1–3 sequences as a reference, they determined that *P. tricornutum* has 4 (possibly 5) BSTs (PtBST1–4), while *T. pseudonana* has 2 BSTs (TpBST1–2). PtBST3 and TpBST2 localized to the PPT within the pyrenoid, while PtBST1, PtBST2, PtBST4, and TpBST1 localized more generally to the chloroplast and immunogold labeling showed more specific localization to the stroma thylakoid membrane surrounding the pyrenoid (Fig. B). In both species, BSTs are upregulated in response to low CO_2_ levels. However, in *P. tricornutum* this is through increased transcription, while in *T. pseudonana* the transcript levels do not change but the proteins are only detectable with immunoblots under low CO_2_ conditions.

The authors made *P. tricornutum* CRISPR-Cas9 nickase knockout-mutants of *PtBST1*, the gene with the strongest transcriptional response under low CO_2_ levels. Under these conditions, the mutants showed a trend toward slower growth than wild type, had lower affinity for dissolved inorganic carbon during photosynthetic O_2_ production (measured by higher K_0.5_, the DIC concentration leading to one-half of the maximum O_2_ evolution rate), and higher nonphotochemical quenching. Under high CO_2_ conditions, there was no difference in these parameters between wild type and mutant. These results suggest that PtBST1 is needed for an efficient CCM and that it also has a role in modulating thylakoid membrane potential and energy balance in the context of photosynthetic electron transport. The HCO_3_^−^ concentration influences H^+^ and pH balance in the chloroplast, where HCO_3_^−^ ions counteract the proton gradient in the thylakoid lumen. These conditions have an effect on light utilization and electron transport in the thylakoid membranes, for instance in the dissipation of excess light energy in nonphotochemical quenching.

The function of diatom BSTs as HCO_3_^−^ transporters demonstrates a convergent evolution compared to what has been shown in Chlamydomonas. Similar to CrBST4 (Adler et al. 2023), PtBST1 has a role not only in facilitating the CCM but also in modulating the ion balance across the thylakoid membrane. However, there is still diversity within common principles; recent discoveries demonstrate that *P. tricornutum* and *T. pseudonana* both have a protein sheath around their pyrenoids that could function as a CO_2_ diffusion barrier (Shimakawa et al. 2023), which is likely performed by other structures in Chlamydomonas, such as a starch sheath (Fei et al. 2022). The current study also highlights the diversity in mechanisms that we see among diatoms, where different BSTs may have their own specific roles and are differentially regulated. We are quickly learning more about the diversity of CO_2_ delivery to pyrenoids and of pyrenoid structures among aquatic organisms.
